# Zones of cellular damage around pulsed-laser wounds

**DOI:** 10.1371/journal.pone.0253032

**Published:** 2021-09-27

**Authors:** James O’Connor, Fabiha Bushra Akbar, M. Shane Hutson, Andrea Page-McCaw

**Affiliations:** 1 Department of Cell and Developmental Biology, Vanderbilt University, Nashville, Tennessee, United States of America; 2 Program in Developmental Biology, Vanderbilt University, Nashville, Tennessee, United States of America; 3 Department of Physics and Astronomy, Vanderbilt University, Nashville, Tennessee, United States of America; 4 Department of Biological Sciences, Vanderbilt University, Nashville, Tennessee, United States of America; 5 Vanderbilt Institute for Integrative Biosystems Research and Education, Vanderbilt University, Nashville, Tennessee, United States of America; 6 Vanderbilt-Ingram Cancer Center, Vanderbilt University, Nashville, Tennessee, United States of America; Fukushima Medical University, JAPAN

## Abstract

After a tissue is wounded, cells surrounding the wound adopt distinct wound-healing behaviors to repair the tissue. Considerable effort has been spent on understanding the signaling pathways that regulate immune and tissue-resident cells as they respond to wounds, but these signals must ultimately originate from the physical damage inflicted by the wound. Tissue wounds comprise several types of cellular damage, and recent work indicates that different types of cellular damage initiate different types of signaling. Hence to understand wound signaling, it is important to identify and localize the types of wound-induced cellular damage. Laser ablation is widely used by researchers to create reproducible, aseptic wounds in a tissue that can be live-imaged. Because laser wounding involves a combination of photochemical, photothermal and photomechanical mechanisms, each with distinct spatial dependencies, cells around a pulsed-laser wound will experience a gradient of damage. Here we exploit this gradient to create a map of wound-induced cellular damage. Using genetically-encoded fluorescent proteins, we monitor damaged cellular and sub-cellular components of epithelial cells in living *Drosophila* pupae in the seconds to minutes following wounding. We hypothesized that the regions of damage would be predictably arrayed around wounds of varying sizes, and subsequent analysis found that all damage radii are linearly related over a 3-fold range of wound size. Thus, around laser wounds, the distinct regions of damage can be estimated after measuring any one. This report identifies several different types of cellular damage within a wounded epithelial tissue in a living animal. By quantitatively mapping the size and placement of these different types of damage, we set the foundation for tracing wound-induced signaling back to the damage that initiates it.

## Introduction

The field of wound repair and regeneration has long sought to connect the signals emanating from wounds to the behavioral changes undertaken by cells around the wound. In response to wounds, tissue-resident cells transition from a stationary and non-proliferative state to a migratory or proliferative state [1–4], whereas immune cells migrate from outside the tissue to clear debris and fight infection by pathogens entering through the wound [5–9]. Ultimately, the signals emanating from wounds must be derived from the damage itself; however, only a few studies have characterized the damaged tissue on a cellular/sub-cellular level to understand the distinct types of damage created by wounds [10–13]. Importantly, our previous studies have found that multiple signaling pathways are initiated around the same time within the same wound, and further, that distinct types of cellular damage initiate each signaling pathway. Specifically, cell lysis leads to the release of cellular proteases, which cleave and activate cytokines in the vicinity of the wound, which in turn signal to surrounding cells through G-protein coupled receptors [14]. However, even before cytokine signaling is evident, cells with a different type of damage–torn plasma membranes–initiate calcium signaling, as extracellular calcium floods in through damaged membranes and out through gap junctions to undamaged neighboring cells [15]. Together, these studies suggest that understanding the origin of wound-induced signaling requires identifying and categorizing different types of cellular damage induced by wounding. In this study, we attempt to solve this problem by classifying various zones of damage around epithelial wounds visualized using genetically-encoded fluorophores, and mathematically defining the relationships between these zones of damage to create a map of the characteristic cellular damage experienced by a tissue following a wound.

It may be impossible to develop a useful map of cellular damage around ordinary trauma wounds experienced in the natural world, as the types of damage would be irregularly arrayed and would not be sufficiently reproducible to identify patterns. However, researchers often employ laser-induced wounding, which has advantages compared to other methods of wounding because of its ability to make reproducible, radially-symmetric, aseptic wounds in a tissue while simultaneously imaging [2,6,15–27]. Two types of lasers are used to damage tissues–continuous wave lasers and pulsed lasers–which make very different types of cellular damage. A continuous wave laser delivers energy to the sample on the order of milliseconds to seconds, and in doing so delivers a high amount of thermal energy to both the ablated region and the surrounding tissue, causing burning and/or explosive ejection of cellular debris from the sample [12]. In contrast, a pulsed laser delivers energy with pulsewidths on the order of femtoseconds to microseconds, dramatically decreasing the thermal energy transfer to the surrounding tissue [12,28]. A pulsed laser superheats only the focal spot, vaporizing it into a bubble of gas known as a cavitation bubble, which expands and contracts within microseconds, causing mechanical (but not thermal) damage to the surrounding tissue [12,13,29,30]. Although continuous lasers were often used through the 1980s, pulsed lasers have become more widely used over the last three decades because of the shorter thermal event, delivering energy so rapidly as to destroy and remove hot tissue before the heat can be transferred, thus reducing thermal damage to the surrounding tissue and removing burn damage as a confounding variable in studying wound signaling and repair [28,29]. Although other methods of wounding have been utilized in wound repair studies, namely puncture [24,31,32], pinch [33,34], scratch wounding [35], or complete amputation [5,36,37], these methods can introduce experimentally-induced variability that hinders reproducibility.

Previously, our labs used pulsed-laser wounding on an epithelial monolayer in living *Drosophila* pupae to understand wound-induced repair signals, specifically how calcium is increased in the cytosol of cells around wounds [15]. Unexpectedly we found that the laser-induced cavitation bubble damages the plasma membranes of epithelial cells around the wound margin, creating microtears that allow extracellular calcium to flood into damaged cells within milliseconds of wounding. To study this phenomenon, we varied the laser energy and found that cavitation bubble area matched the areas of extracellular calcium entry, membrane depolarization, and extracellular dye entry, three indicators that plasma membrane integrity was lost in the tissue damaged by the cavitation bubble [15]. Many of the cells with plasma membrane damage were able to repolarize and survive, demonstrating that not all damaged cells die. Other reports have demonstrated loss of plasma membrane integrity of cells around wounds made by multiple types of injury [11,17,27,35,38,39]. Indeed, in these studies many of these cells with plasma membrane damage survived and were able to restore membrane integrity [11,17]. Thus, these previous studies show there are *at least* two populations of cells that experience damage following a wound: cells near the center of the wound that are damaged so severely that they are destroyed and cells distal from the center of the wound that are damaged but ultimately survive and participate in the repair process. In this study, we identify several more types of cells that are damaged by laser wounds; specifically, we monitored initial laser-induced rupture, delayed cell lysis, nuclear membrane damage, plasma membrane damage, chromatin disruption, Ecadherin loss, and a calcium expansion outward from the wound site. The severity of damage was inversely correlated with radius from the wound center, and by varying wound size, we find that these regions of damage are arrayed in predictable and reproducible patterns around the wound.

## Results

**Fig 1:**
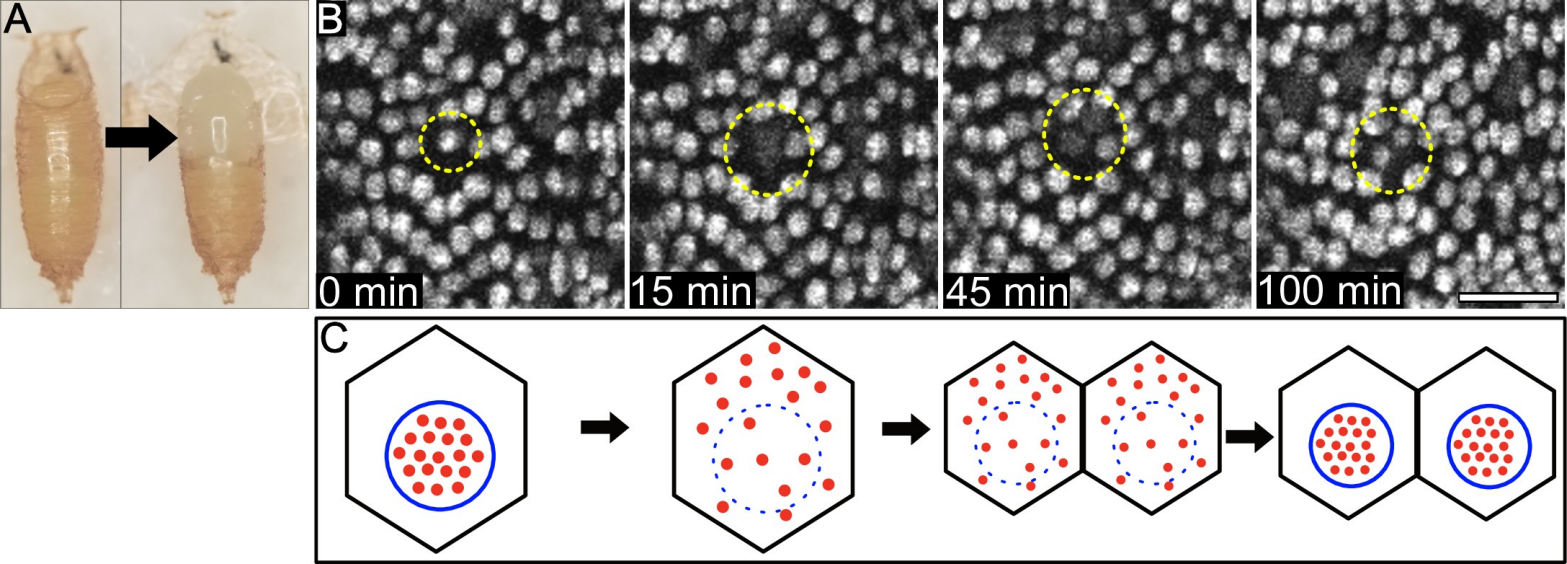
mCherry.NLS reports nuclear envelope integrity. (A) An intact *Drosophila* pupa (left) and one prepared for imaging with the pupal case partially removed (right), exposing the pupal notum. (B) Interphase nuclei in unwounded tissue are labeled by mCherry.NLS driven by the *Gal4/UAS* system. During mitosis, the fluorescence signal evident at 0 min disperses when the nuclear envelope breaks down, seen within circles in panels at 15 min and 45 min, before reappearing as two distinct/punctate nuclei at 100 min. (C) A schematic showing the diffusion of mCherry.NLS out of the nucleus during mitosis and its concentration in nuclei again after the nuclear envelope reforms in the daughter cells. Thus, the localization of mCherry.NLS reports nuclear membrane integrity. Scale bar = 25 μm.

We chose *Drosophila melanogaster* (fruit flies) for wounding studies because they are genetically tractable, with numerous existing strains expressing genetically-encoded fluorescently-tagged proteins easily visualized during live imaging. We analyzed wounds in the pupal notum, the epithelial tissue on the dorsal thorax of the pupa, easily accessible after partially removing the pupal case [16] (Fig 1A). Wounds were administered by pulsed-laser ablation, allowing simultaneous wounding and imaging *in vivo*. Because the pupa is stationary, it can be easily mounted, wounded, and imaged for extended periods of time (hours to days) and still survive to adulthood.

Using the *Gal4/UAS* system [40], we drove expression in the pupal notum of the fluorescent protein mCherry with a nuclear localization signal (mCherry.NLS) to visualize live epithelial nuclei. In unwounded animals, mCherry.NLS fluorescence is normally punctate but becomes diffuse during cell division, later re-appearing as two separate punctae (Fig 1B). Since mCherry.NLS is a freely-diffusible fluorescent protein carrying a nuclear localization signal, this pattern indicates that mCherry.NLS diffused into the cytosol during nuclear membrane breakdown (Fig 1C).

**Fig 2:**
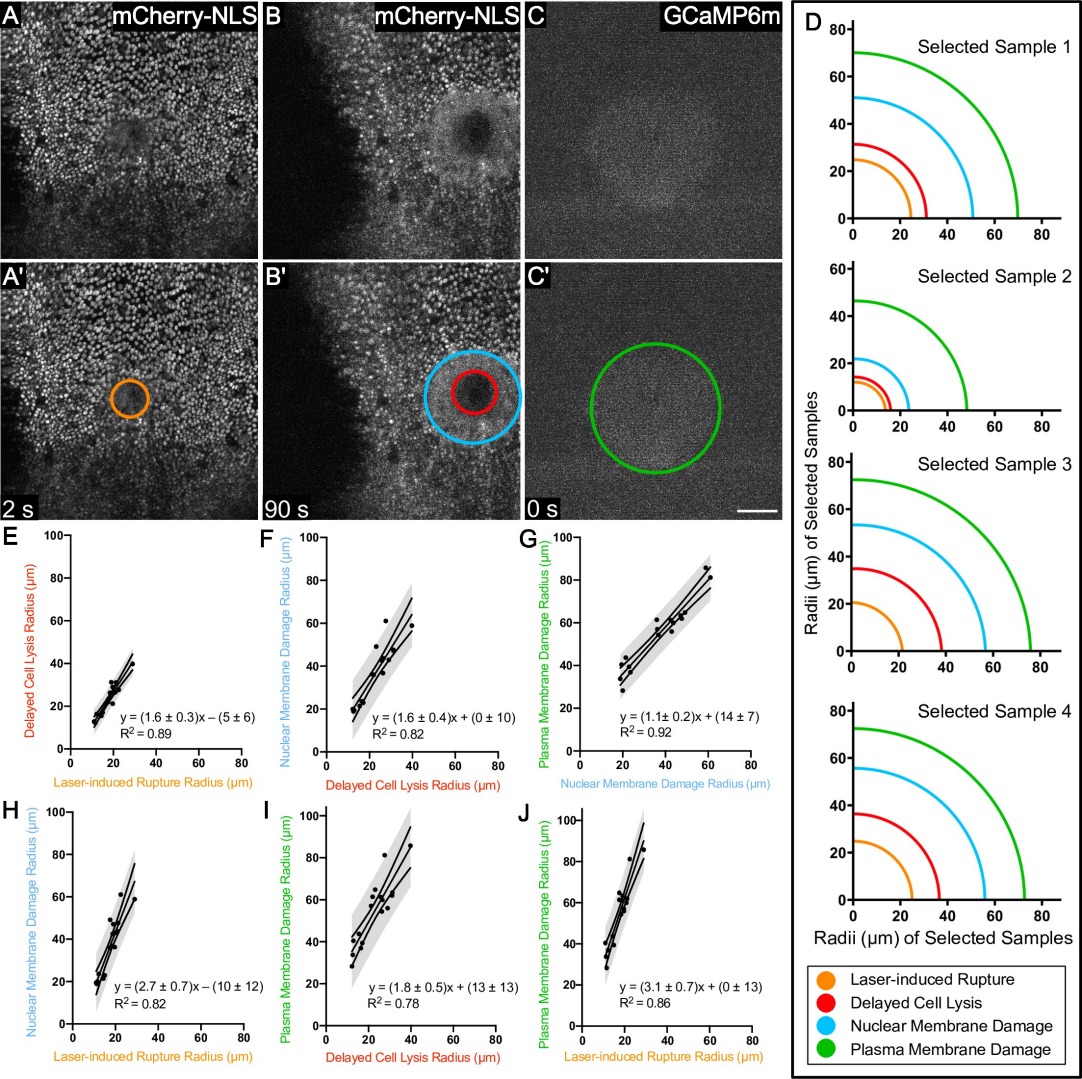
Several zones of damage are evident around pulsed-laser wounds. (A) The region of laser-induced rupture is observed as the area of disrupted mCherry.NLS within the first two frames after wounding (here 2 seconds), annotated with an orange circle in A’. (B) The region of delayed cell lysis is observed as the complete loss of mCherry.NLS at 90 seconds, (red circle in B’) and the region of nuclear membrane damage is observed as a diffuse, non-nuclear mCherry.NLS signal (light blue circle in B’), maintained within the cells by the plasma membrane at 90 seconds after wounding. (C) The region of plasma membrane damage is observed as an increase in cytoplasmic calcium levels immediately after wounding (green circle in C’, as reported by GCaMP6m, a fluorescent calcium indicator, in the first frame after wounding (here 0 seconds). In this region, the wound creates microtears in the plasma membrane, allowing immediate influx of extracellular calcium. All images are of the same wound; the frame is shifted in B,B’. (D) Actual measurements of radii in four different samples demonstrates a consistent relationship between these regions; as any one of these regions increases in radius, the others likewise increase in radius. Panels E-J quantify this trend with 95% prediction bands. n = 17 pupae. Scale bar = 50 μm.

Upon pulsed-laser ablation, the mCherry.NLS fluorescence was lost at the wound center within the first two frames after ablation (usually ~2 seconds). Because the fluorophore was lost from both the nucleus and the surrounding cytoplasm (orange circle, Fig 2A’), this loss of fluorescence indicates cell destruction and rupture. We term this immediate loss of mCherry the **region of laser-induced rupture.** This region includes the area of the laser focus and plasma formation where photochemical damage destroys macromolecular structures (~ 1 μm in diameter, [13]) as well as the area of the most severe cavitation-driven shear stress, which causes immediate cellular destruction. Note that the region of laser-induced rupture is nonetheless much smaller than the maximum area of the cavitation bubble (see below) [13,41]. In the wounds made for this study, the size of laser-induced rupture ranged between 10 and 30 μm in radius.

Within 90 seconds after pulsed-laser ablation, the mCherry.NLS signal changed in two distinct ways. First, the region devoid of mCherry signal increased in size, indicating that even after ablation, cells continue to lyse (compare Fig 2A and 2B). No fluorescence signal returned to this region until late in the repair process when distal cells migrated in to repair the wound. This larger area of cell death we term the **region of delayed cell lysis** based on the complete loss of mCherry.NLS by 90 seconds after wounding (red circle, Fig 2B’). Both the initial laser energy and the ensuing cavitation bubble may contribute to cell lysis in this area.

Second, beyond the delayed cell lysis area, the mCherry.NLS became diffuse, no longer confined to the nucleus but still remaining in the tissue (light blue circle, Fig 2B’), similar to its appearance during mitosis (Fig 1B). Interestingly, later in the repair process the mCherry signal returned to the nucleus in some of these cells, indicating that some of these cells survive (S1 Fig). We concluded that these cells have undergone damage to the nuclear membrane, causing the previously nuclear-localized mCherry to be released from the nucleus into the cytosol (Fig 2B). We term this the **region of nuclear membrane damage**.

To understand how these zones of damage compared with other types of damage we had already characterized, we compared the mCherry.NLS patterns with those of GCaMP, a ubiquitously expressed genetically encoded cytoplasmic calcium reporter. Our previous work demonstrated that within the first frame after pulsed-laser ablation, cells near the wound exhibit an increase in GCaMP fluorescence. This region of immediate GCaMP fluorescence indicates the region where the plasma membrane is torn by the laser-induced cavitation bubble, allowing the rapid influx of extracellular calcium [15]. The cavitation bubble expands and collapses within microseconds, and extracellular calcium enters through microtears in the plasma membrane within ~10 milliseconds, increasing its concentration over the next few seconds as more calcium enters the cell [15]. Thus, the immediate GCaMP signal indicates the **region of plasma membrane damage** (green circle, Fig 2C’). Although the GCaMP signal is still dim in the first frame after wounding, the first frame provides the most accurate measure of the area of plasma membrane damage because the calcium diffuses outward to neighboring cells through gap junctions over the next 15–20 seconds, enlarging the area of GCaMP6m fluorescence beyond the region of plasma membrane damage [15].

After measuring the radii of these four well-defined regions in multiple samples with varying wound sizes, we found the four regions maintained the same ascending size order within every sample: Laser-induced rupture < delayed cell lysis < nuclear membrane damage < plasma membrane damage (Fig 2D). To determine whether there was a consistent relationship between the radii of each region, we analyzed how each of these four regions related to one another in wounds of various sizes and graphed the six relationships (Fig 2E–2H). This analysis revealed that the radius of each zone was linearly correlated with the radius of each other zone, with an R^2^ value for each of the trendlines lying between 0.78 and 0.92. Importantly, these results show that each laser-induced wound creates reproducible regions of damage around a single wound, and all of these regions can be reasonably estimated after measuring the radius of a single one. Not only were adjacent zones of damage related linearly, but also the linear relationship was maintained even between laser-induced rupture and plasma membrane damage, the smallest and the largest zones of damage respectively (Fig 2J).

**Fig 3:**
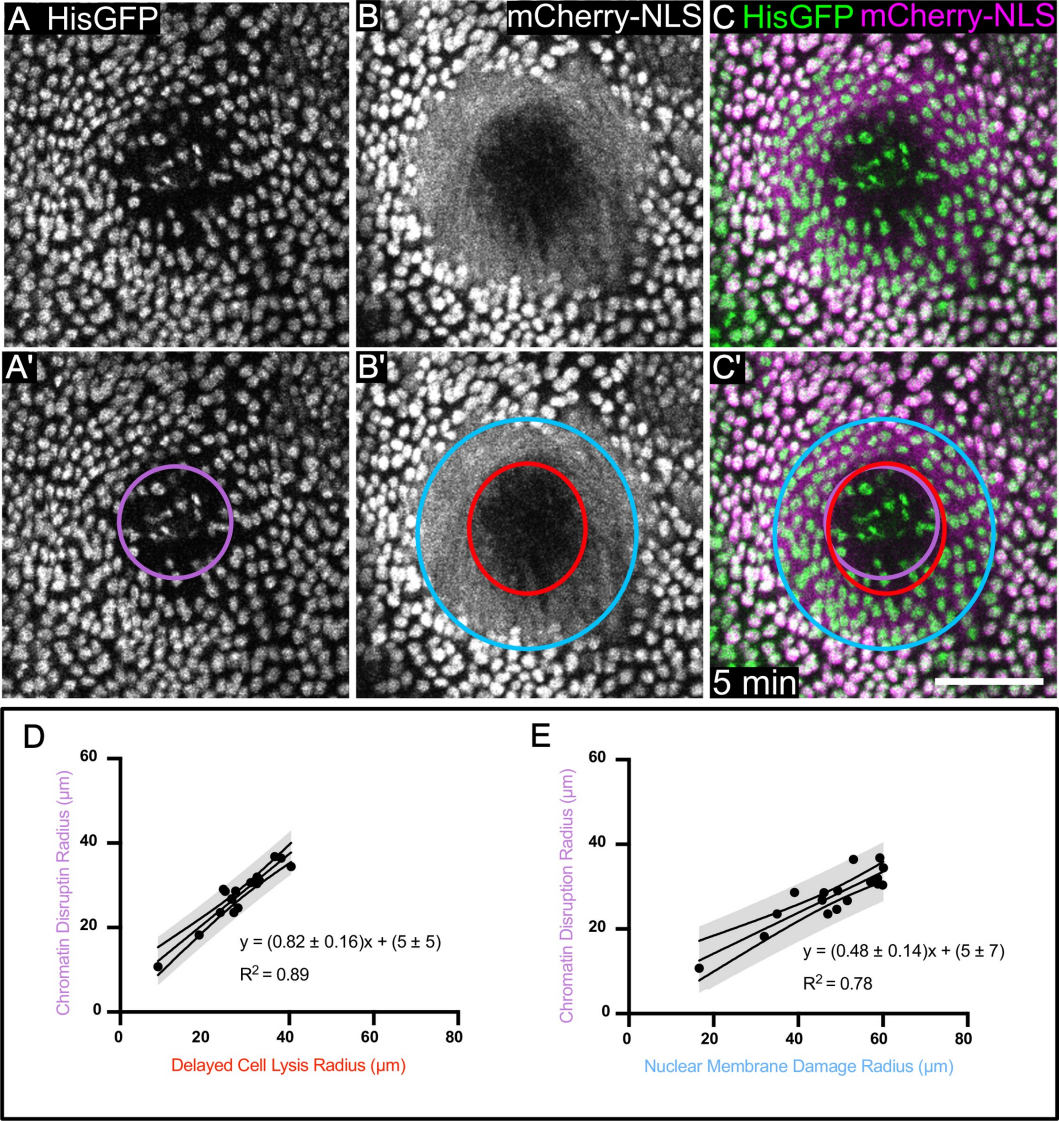
Histones reveal that within the region of nuclear membrane damage, some nuclei have intact chromatin whereas nuclei closer to the center have disrupted chromatin. (A) His2Av-GFP, which labels histones in chromatin, reveals a damage region with misshapen chromatin and decreased fluorescence, indicated with a purple circle in A’. (B) mCherry.NLS in the same wound reveals the region of nuclear membrane damage, (blue circle in B’) and the region of delayed cell lysis (red circle in B’). (C) The overlay of His2Av-GFP with mCherry.NLS shows that the region of chromatin disruption is much smaller than the region of nuclear membrane damage, but similar in size to the region of delayed cell lysis. (D-E) The relationships between the region of chromatin disruption and the regions of nuclear membrane damage (D) and of delayed cell lysis (E) are linear. The 95% confidence interval is indicated. n = 17 pupae. Scale bar = 50 μm.

To better understand the nuclear membrane damage, we co-expressed mCherry.NLS and a GFP-tagged histone variant, His2Av-GFP. Unlike mCherry.NLS, the GFP-tagged histone is a fusion protein incorporated into chromatin, regardless of cell cycle stage or nuclear envelope integrity. Therefore, we could track nuclear destruction independently of nuclear membrane damage. At 5 minutes after wounding, there were many intact, well-ordered, His2Av-GFP-containing nuclei within the region of nuclear membrane damage, confirming that the nuclear membrane, but not necessarily chromatin, are damaged in this region (Fig 3A–3C). However, toward the center of the wound there were some misshapen punctae of His-GFP (Fig 3A–3C). We term this the **region of chromatin disruption**, which we measured and compared to the regions of delayed cell lysis and nuclear membrane damage (Fig 3A–3C). We found that the chromatin disruption region was much smaller than the nuclear membrane damage region (Fig 3D) and was similar in size to the region of delayed cell lysis (Fig 3E). These results demonstrate that even after cell lysis, the His-GFP and chromatin do not freely diffuse. Further, they raise the possibility that delayed lysis and chromatin disruption are related. Although the best-fit equation relating these two zones had a slope less than one (Fig 3D), a simple y = x relationship between the regions of chromatin disruption and delayed cell lysis had an R^2^ of 0.84, which shows the two regions indeed correlate very well with each other.

**Fig 4:**
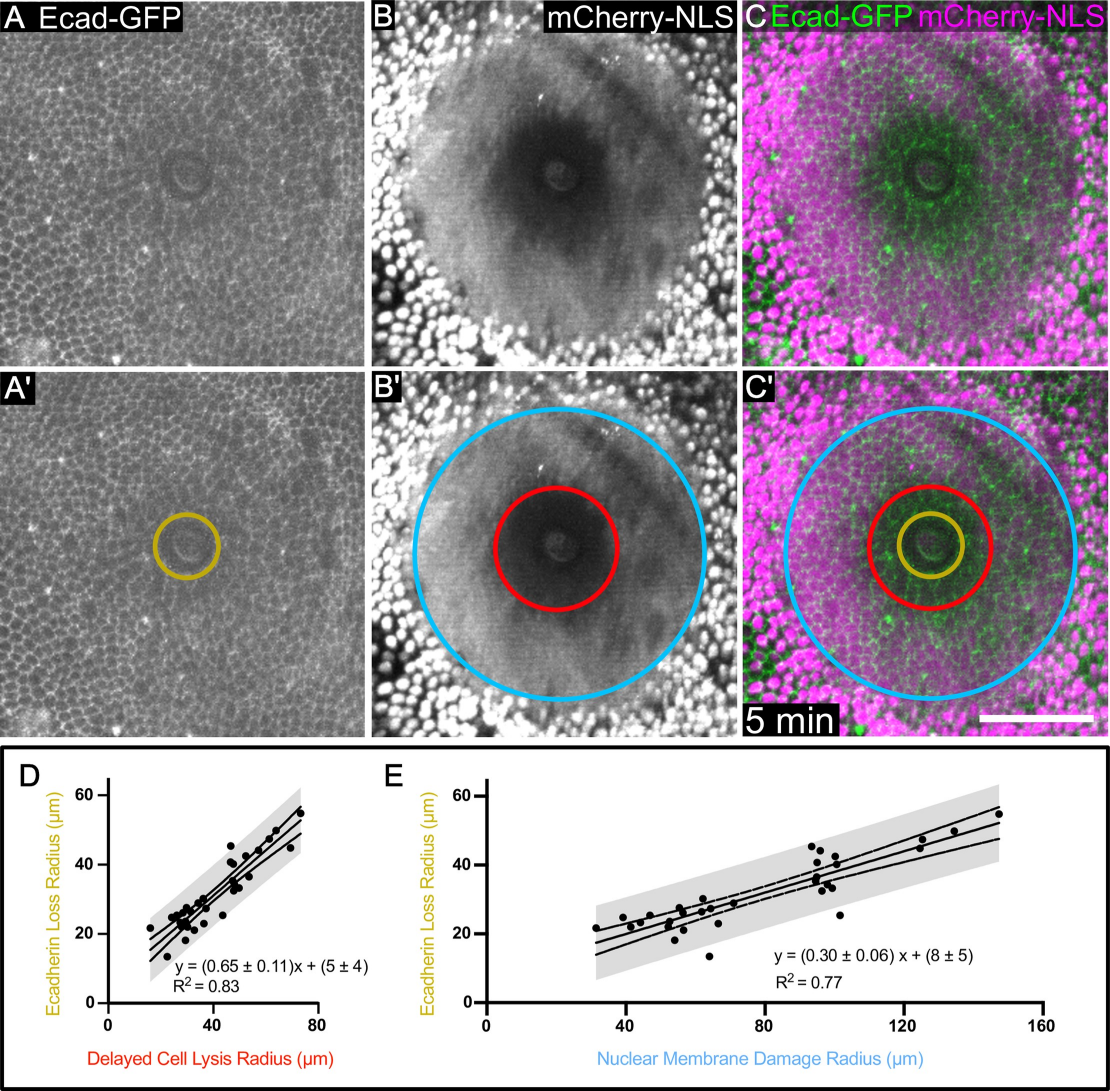
Cell borders are disrupted following wounding. (A) Genetically encoded Ecadherin-GFP marks cell borders and reveals damage around a pulsed-laser wound. The region of immediate Ecadherin-GFP loss is indicated with a gold circle in A’. (B,C) The region of immediate Ecadherin loss is distinct from and within the regions of delayed cell lysis (red circle) and nuclear membrane damage (blue circle). (D) The relationship of the radius of delayed cell lysis to the radius of immediate Ecadherin loss is linear. (E) The relationship of the radius of nuclear membrane damage to the radius of immediate Ecadherin-loss is linear. 95% prediction interval is indicated in D,E. n = 32 pupae. Scale bar = 50 μm.

To monitor individual cells in addition to individual nuclei, we co-expressed the mCherry.NLS fluorescent marker with GFP-tagged Ecadherin to visualize nuclei and cell borders simultaneously. Interestingly, a new region of damage was evident after wounding as a region of **immediate Ecadherin loss**. This region was evident starting at the first frame after wounding, although we often measured it at 5 min after wounding as the radius of clear Ecadherin loss does not significantly change within this time (S2 Fig), and because this allowed time to switch the objective from the 40x ablation objective to a 63x objective for higher-resolution imaging. The Ecadherin labeling along cell borders appeared disrupted within a radius smaller than the region of nuclear membrane damage (yellow and light-blue circles respectively, Fig 4A–4C and 4E), and surprisingly, smaller than the region of delayed cell lysis where cells had completely lost mCherry.NLS signal (yellow and red circles respectively, Fig 4A–4D). The stability of Ecadherin complexes in adherens junctions at least transiently withstood damage such as cell lysis and release of cytoplasm, but over the next hour, the region of Ecadherin loss expanded (S1 Movie, S2 Fig). This latter gradual loss of Ecadherin may be similar to the endocytosis of Ecadherin observed at the margin of wounds in fly embryos [42,43], but endocytosis is not fast enough to drive the immediate loss of Ecadherin evident as soon as the first frame after wounding (S1 Movie, S2 Fig).

Given that Ecadherin may remain for some time along the borders of lysed cells and that it may be endocytosed and removed from the borders of cells that are nonetheless intact, we wanted to determine the relationship between the gradual loss of Ecadherin and cell lysis. To do so, we imaged pupae co-expressing Ecadherin-GFP and mCherry.NLS for 90 minutes following wounding. Many adherens junctions that initially appeared as intact cell borders started breaking down around 30 minutes and were gone by 90 minutes (S2 Fig, S1 Movie), disassembling throughout the region of delayed cell lysis more or less simultaneously. The region where Ecadherin complexes were dismantled (S2 Fig, yellow asterisks) spanned the entirety of the delayed cell lysis region determined by mCherry.NLS (S2 Fig, red circle) as well as one to two cell diameters into the region of nuclear membrane damage (S2 Fig, light blue circle). Thus, the Ecadherin/adherens junctions within the delayed cell lysis region that appear stable five minutes after wounding represent the carcasses of dead/dying cells that do not survive the repair process.

**Fig 5:**
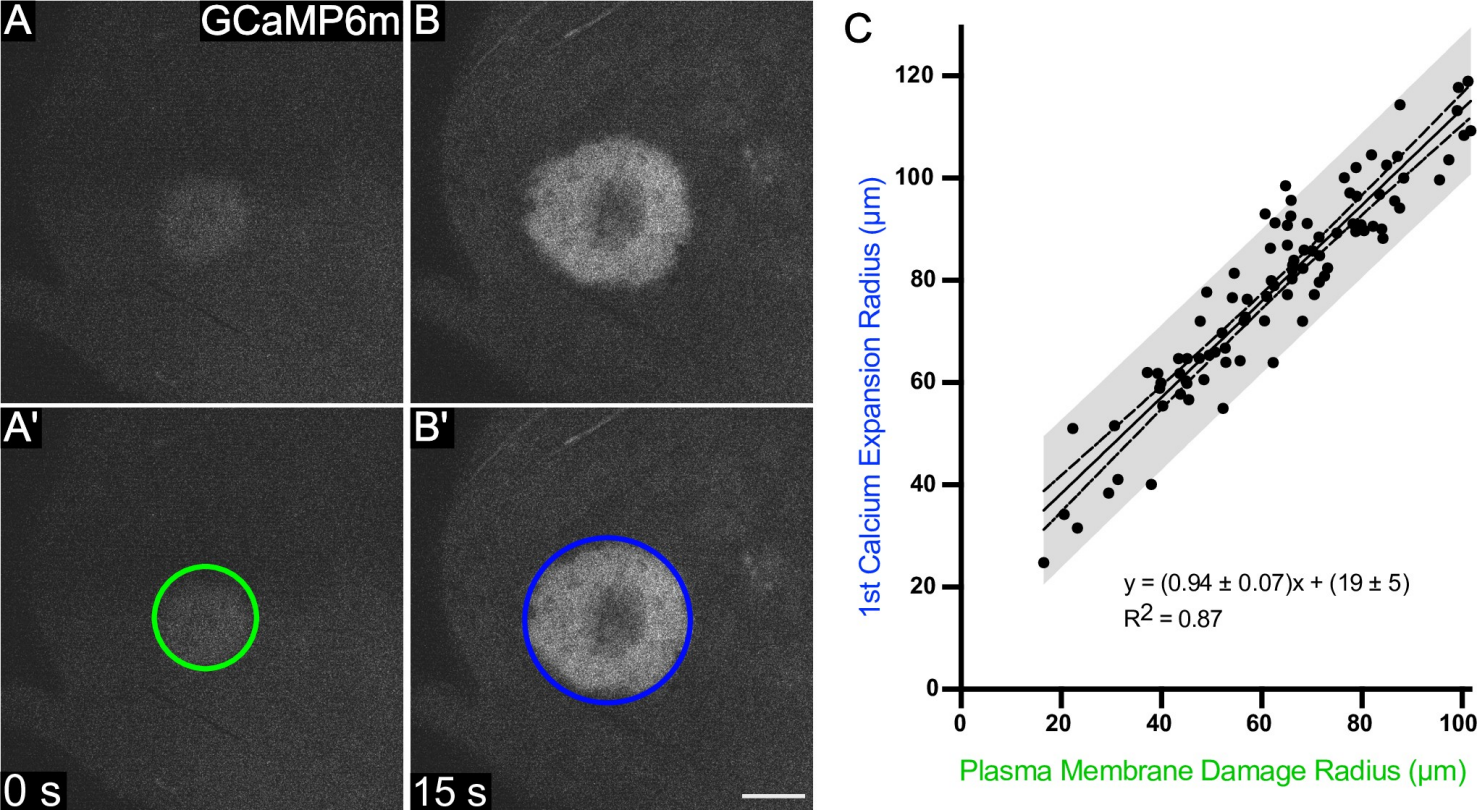
The first expansion of calcium extends about 20 μm from the region of plasma membrane damage, independent of wound size. (A) The influx of calcium immediately after wounding identifies cells with plasma membrane damage. This region is observed by an immediate increase in GCaMP fluorescence, indicated by a green circle in A’. (B) The calcium radius increases over the next ~15 seconds [15], and the maximum first expansion region is indicated with a dark blue circle in panel B’. The relationship between the plasma membrane radius and the first expansion radius is linear. Because the slope is ~1, the y-intercept of 19 indicates that the 1^st^ expansion radius is expected to be ~20 μm larger (~2 to 4 cell diameters) than the radius of plasma membrane damage, regardless of wound size or laser energy. n = 92 pupae. Scale bar = 50 μm.

The last damage-induced landmark we analyzed was the progression of the cytoplasmic calcium signal outward from the wound within the first seconds after wounding, measured as an increase in the radius of GCaMP fluorescence. After extracellular calcium enters cells through plasma membrane damage, that initial influx of calcium expands outward to neighboring cells through gap junctions within 15 seconds following wounding [14,15]. We have termed this the **first calcium expansion**, so called because there is an independent second calcium response later [14]. Although the first calcium expansion does not represent a type of cellular damage, it may represent damage-induced information transmitted to nearby cells, and we were interested to see how it compared in size to the damage itself. We compared the radius of the first calcium expansion to the radius of plasma membrane damage and found that it was positively correlated with a trendline of slope near 1, but with a y-intercept of 19 μm (Fig 5). This linear relationship suggested that regardless of wound size, the initial influx of calcium travels about 20 μm outward to the next 2–4 neighboring cells, which experience this wound induced calcium signal even though they are ostensibly undamaged.

**Fig 6:**
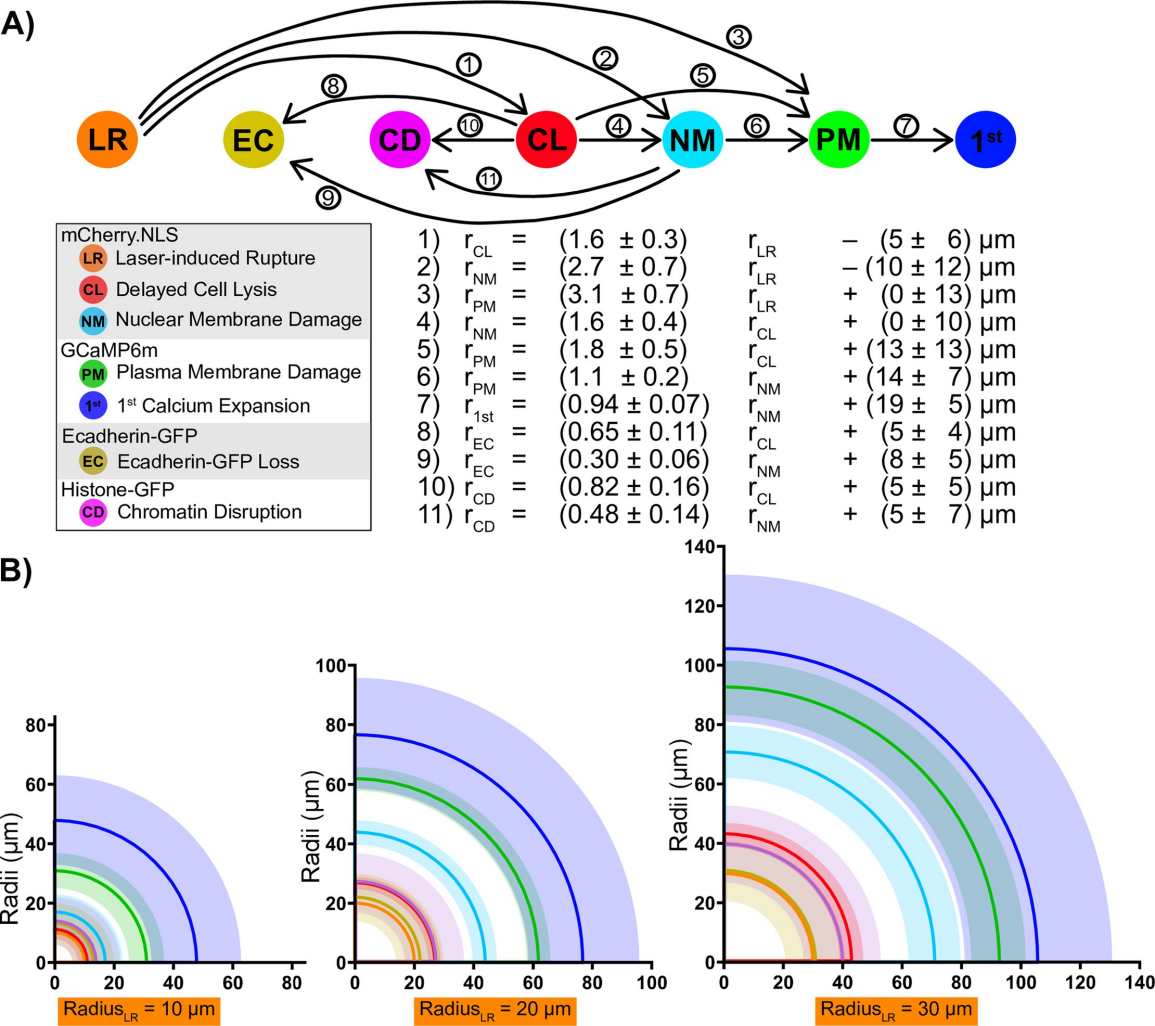
Each zone of damage can be estimated given one of them. (A) Schematic of how to calculate each zone of damage value from a given one, with each of the eleven linear equations displayed below corresponding to the arrows above. Arrows go from independent variable (x) to dependent variable (y), corresponding to Eqs 1–11 derived in Figs 2–5. The complete set of 42 equations necessary to relate each of the 7 zones of damage were derived according to the Materials and Methods and are provided in S2 Dataset. (B) Three hypothetical wounds are indicated, each with a different initial laser-induced rupture radius (10, 20, or 30 μm). The equations in S2 Dataset were used to derive the radii of the six other zones of damage from the given laser-induced rupture radius. The 95% confidence intervals are displayed for all values. With a radius of 10 μm, the first four regions overlap (laser-induced rupture, immediate Ecadherin loss, chromatin disruption, delayed cell lysis). Because the average cell diameter in this tissue is ~7 μm, the overlap suggests that these four regions may be within one cell diameter. As the wound gets larger, the regions become more distinguishable.

By analyzing these fluorescent proteins, we have identified seven concentric zones of damage surrounding a single laser wound. These zones of damage are linearly related to one another by the 11 equations in Figs 2–5 derived from the observed data (observed data is provided in S1 Dataset). Further, these 11 equations can be combined to relate any two zones of damage (Fig 6A; see Materials and Methods), allowing a reasonable estimation of the radius and 95% confidence interval of all the zones of damage from any one of them. To do this, we provide an Excel book (S2 Dataset) that describes the 42 linear equations required to approximate any of the zones of damage from a measurement of a single zone (sheet 1: Total Equations List). Further, seven subsequent Excel sheets are provided to allow the user to input a radius of one zone of damage and output the approximate radius and confidence intervals for the remaining six zones of damage (sheets 2–8). As an example, we calculated these damage regions for laser-induced rupture radii of 10, 20, or 30 μm, corresponding to small, medium, or large wounds made in this study (Fig 6B). In doing so, we found the radii of the four inner-most regions of damage were clustered extremely tightly together in small wounds (10 and 20 μm laser-induced rupture), such that their radii are less than a single cell diameter (~7 μm) apart from each other. Thus, around small wounds, it is not meaningful to distinguish those four different regions of laser-induced rupture, immediate Ecadherin loss, chromatin disruption, and delayed cell lysis. However, in larger wounds with greater than 20 μm of laser-induced rupture, the seven regions of damage and wound signaling separate from each other and resolve into the same ascending size order: laser-induced rupture < immediate Ecadherin loss < chromatin disruption ~ delayed cell lysis < nuclear membrane damage < plasma membrane damage < first calcium expansion.

## Discussion

These data show that a wound induced by pulsed-laser ablation causes tissue damage in a graded pattern and not in a uniform manner. Damage is most severe at the center of the wound, which is the focal point of the laser, and is progressively less severe with distance from the center. Importantly, cellular damage encompasses a region much larger than the area where cells are entirely destroyed, and many damaged cells recover and participate in wound repair.

From a spatial perspective, the zones of damage are arranged in a fixed order. Laser wounding involves a combination of photochemical, photothermal, and photomechanical effects, each falling off at a different rate with distance. Each type of damage is likely triggered by one or more particular effect at specific energy or stress thresholds. The first and most central visible zone of damage is the immediate **laser-induced rupture** of cells. At the very center of the wound, the highest laser power drives multiphoton ionization, plasma formation and ultimately molecular recombination that destroys biomolecules [41]. Just slightly further out, stresses from the rapidly expanding cavitation bubble and shock wave destroy macromolecular assemblies. Together these types of damage would appear as a region devoid of mCherry.NLS fluorescence in the first frame after wounding. This region of immediate laser-induced rupture may be the same as the region of **immediate Ecadherin loss**; perhaps the loss of Ecadherin represents one specific type of macromolecular destruction. Although both these regions are recognized by a loss of fluorescence, this loss is more than photobleaching. We note that some molecules retain fluorescence within this area, such as histone-GFP, which retains its fluorescence even though the morphology is disrupted (discussed below).

Moving outward from the center of the wound, the next region of damage is the region of **delayed cell lysis**, identified by the area that loses mCherry.NLS fluorescence in the minutes after wounding. We had expected cell lysis to be an immediate response to damage, but it appears instead that damage triggers lysis in many cells over the course of minutes [14]. It is noteworthy that the region of **immediate Ecadherin loss** is smaller than the region of delayed cell lysis within the first five minutes after wounding. A potential explanation is that Ecadherin is present in stable macromolecular complexes at the junctions, and therefore may appear intact even if the corresponding cell has lysed. Interestingly, Ecadherin appears to be disassembled over a much larger area during the next 90 minutes, with disassembly happening around the lysed cells and intact borders remaining only around cells that survive and participate in the wound response (S2 Fig). Previous studies in embryos have reported that Ecadherin is rapidly endocytosed from the wound margin within minutes of wounding, and the endocytosis of adherens junctions is essential for building the actin-myosin purse-string [42,43]. The loss of Ecadherin from the proximal border of the closest surviving cells in pupal wounds could be a similar response; however, the earliest loss we observed is distinct, as are the losses over the next hour from cells that lyse (S1 Movie). Our results raise the possibility that in addition to functional remodeling of the cell-cell junctions that occurs in cells that participate in the repair process, Ecadherin-GFP removal after wounding may also represent the removal of cellular debris.

The region of delayed cell lysis largely overlaps with the region of **chromatin disruption**, assessed after lysis. It may be that chromatin appears disrupted because a cell no longer protects it; conversely, perhaps damage to the chromatin propels cell lysis. The question of whether the regions of delayed cell lysis and chromatin disruption represent the same region brings up the issue of the resolution of detection. Although the damage profile may cause a gradient in damage to molecules, our limit of detection is a single cell, with a diameter of about 5–10 μm. Thus, two regions of damage that differ by less one-cell diameter cannot be distinguished and may have no functional difference for the tissue response to the wound. From this perspective, it may be useful to consider the regions of delayed cell lysis and His-GFP disruption to be the same.

The next distinct zone is **nuclear membrane damage**, identified by the cloud of mCherry.NLS that leaves the nuclear compartment and floods the cytoplasm when laser-induced stresses compromise nuclear membrane integrity. In addition to nuclei, we expect other membrane-bound organelles to be damaged by similar amounts of stress in this same region. In the more central zones of laser-induced rupture/immediate Ecadherin loss and delayed cell lysis/His-GFP disruption, all the cells die. In contrast, in the zone of nuclear membrane damage, some cells die but most cells recover and participate in wound closure (S1 and S2 Figs). The actomyosin purse-string that forms at the wound margin likely segregates the cells that will survive and repair from those that will die [1,2,16].

The most distant zone of cellular damage is the zone of **plasma membrane damage**. In this region, the mechanical shear force of the cavitation bubble rips the plasma membrane, and this zone is identified by the immediate GCaMP fluorescence from extracellular calcium flooding into the cytoplasm. In a previous study, we analyzed this zone extensively [15] and found that it correlates with both an area of membrane depolarization and area of permeability to extracellular dyes. On kymographs, we visualized calcium entering into cells through distinct spots within this region. Cells with damaged plasma membranes in this region are able to repair the damage; indeed, the extracellular calcium is probably an important trigger for initiating plasma membrane repair [17,18]. As we demonstrated previously, the influx of calcium is not contained within the damaged cells, and calcium expands through gap junctions to neighboring cells in a process we call the **first calcium expansion**. Although not technically a zone of damage, this landmark is caused by damage. Interestingly, the first expansion was a constant distance, about 20 μm, out from the region of plasma membrane damage. This result implies that the first expansion is entirely determined by the initial influx, and the 20 μm distance is likely determined by the kinetics of calcium buffering and re-uptake systems.

The radii of the regions of cellular damage are linearly related, in surprisingly simple relationships that we did not expect at the outset. We derived equations relating the regions of damage, and we expect these equations will be useful in estimating each region of damage when only one is known. For example, any freely diffusing fluorescent molecule expressed in cells can reveal the area of initial laser rupture and/or delayed cell lysis, depending on the timing of data acquisition; from either of these data points, estimates for the regions of stable protein complex destruction (Ecadherin loss), nuclear membrane damage, and plasma membrane damage, and the region of high initial calcium can all be calculated.

From a temporal perspective, the initial laser pulses and following cavitation bubble cause immediate and complete cellular destruction at the very center of the wound, and simultaneously they cause a loss of nuclear membrane integrity in cells farther out, and a loss of plasma membrane integrity in cells still farther away. Next, in the milliseconds to seconds following wounding, the first known intercellular wound signal–calcium–floods into damaged cells and then travels to neighboring cells, notifying unwounded nearby cells of the damage that has just occurred. Finally, over the next minute or two, a region of delayed cell lysis appears around the initial laser-induced rupture, where cells lose their cytoplasmic contents.

It is interesting to speculate whether each zone of cellular damage evaluated here might correspond to a specific downstream repair behavior initiated by the organism in response to the wound. For example, the delayed cell lysis region may be associated with a region of protease release and/or ligand activation leading to a GPCR-mediated calcium response in the minutes following a wound [14]. The zones of calcium signaling may be similar to the zones of increased reactive oxygen species [5,6,44] and/or actin remodeling [16,18]. The debris left behind in the center of wound and visualized in Histone-GFP and Ecadherin-GFP samples may represent a type of debris that is cleared away by immune cells later in the repair process [45–47].

The results of this study expand on an earlier study by Hellman, et al. [10], which identified 3 separate regions of damage after laser wounding in cell culture: a region of destruction at the center, a necrosis region of cells that die progressively over time, and a region of “molecular delivery” whereby wounded cells become permeable to the extracellular space. These seem qualitatively similar to our identified regions of laser-induced rupture, delayed cell lysis, and plasma membrane damage, respectively, which here we have shown in a living animal. Additionally, we report a nuclear membrane damage region and characterize sub-cellular damage markers using genetically-encoded fluorophores. Whereas Hellman, et al. analyzed how regions of damage varied with laser energy [10], our focus is on how the regions of damage correlate with each other, with the goal of estimating the distinct regions for any pulsed-laser wound.

## Conclusions

This study analyzed wounds made by pulsed-laser ablation, which creates reproducible and orderly wounds, with the regions of damage arranged in a reproducible manner. In trauma wounds caused by puncture, crush, or pinch wounds, all the types of damage examined in this study are expected to occur but without precise spatial patterning, rendering them difficult to identify and analyze. Thus, pulsed-laser ablation is a valuable research tool, allowing distinct regions of damage to be resolved through microscopy, and offering the opportunity to relate each type of damage with specific cellular responses.

When an epithelial barrier is breached, the cellular landscape is dramatically altered and must be repaired to avoid exposure to pathogens or loss of internal fluid [24,48]. Although some cells at the center of a wound may be destroyed immediately or lost progressively, many partially damaged cells around the wound survive and respond by initiating the repair process, indicating that both cellular repair and tissue repair programs are activated by the same wound [4,16,39]. Undamaged cells farther out also receive instructive cues from the wound, recruiting them to participate in the repair process as well [4,16,49,50]. To understand how damage initiates repair, it is critical to understand the types of damage present around a wound. However, little work has been done to characterize damaged tissue on a cellular/sub-cellular level and understand how the epithelial architecture is altered in the immediate aftermath of wounding. By characterizing zones of cellular damage, we have provided insight into what kinds of cellular changes occur to the tissue in the moments following wounding. As we have recently reported, some of these specific regions of damage initiate specific signals, and probably more have yet to be discovered. Ultimately, cellular damage itself acts as an input stimulus for the eventual behavioral output. Future studies will explore how these damage-induced cellular alterations initiate the repair process.

## Materials and methods

### Fly lines

Drosophila crosses were maintained at 18°C for 2 days then progeny transferred to 29°C for 4–5 days before wounding. In Figs 2 and 5, calcium was analyzed in flies heterozygous for *ActinP-GCaMP; pnr-Gal4*, *UAS-mCherry*.*NLS*, *tubP-Gal80*^*ts*^. In Fig 3, histones were analyzed in flies heterozygous for *His2Av-EGFP; pnr-Gal4*, *UAS-mCherry*.*NLS*, *tubP-Gal80*^*ts*^. In Figs 4 and S2, cell borders were analyzed in flies heterozygous for *Ecadherin-GFP; pnr-Gal4*, *UAS-mCherry*.*NLS*, *tubP-Gal80*^*ts*^. Unique identifiers and sources are *Pnr-Gal4* (FBti000401), *UAS-mCherry*.*NLS* (FBti0147460), *tubP-Gal80*^*ts*^ (FBti0027797), *His2Av-EGFP*.*C* (FBst0024163), *Ecadherin-GFP* also known as *Ubi-p63E-shg*.*GFP* (FBti0151829). All flies were derived from progenitor stocks housed at Bloomington Drosophila Stock Center, except for Ecadherin-GFP from the Kyoto Stock Center, and ActinP-GCaMP6m, made by our lab and reported in O’Connor et al, 2021 [14].

### Pupal mounting

Pupal mounting was performed as described previously [15]. White prepupae were identified and aged for 12–18 hours After Puparium Formation (APF) at 29°C. Multiple pupae were placed on a piece of double-sided tape (Scotch brand, catalog #665), ventral side down on a microscope slide, and their anterior pupal cases were removed with fine tipped forceps to reveal the notum epithelium (as in Fig 1A). The entire piece of double-sided tape with dissected pupae was gently lifted from the microscope slide and adhered to a 35 mm x 50 mm coverslip (Fisherbrand, cat#125485R) so that the pupal nota were laid against the coverslip, with the pupae between the coverslip and the tape layer. Then, an oxygen permeable membrane (YSI, standard membrane kit, cat#1329882) was applied over the pupae and secured to the coverslip with additional double-sided tape so pupae would not become dehydrated or be deprived of oxygen.

### Live imaging

Live imaging of pupae was performed using a Zeiss LSM410 raster-scanning inverted confocal microscope with a 63X 1.4NA or 40X 1.3 NA oil-immersion objective. Raster-scans were performed with a 2.26 seconds scan time per image with no interval between scans. All images are single optical slices in the z-plane of greatest signal clarity, with the exception of Ecadherin-GFP images collected for Fig 4, which were Maximum Intensity Projections of 5–7 slices separated by 1 μm each, representing a z-depth of 5–7 μm. All radial analysis was performed on single optical slices, with the exception of the radius of Ecadherin loss which analyzed said maximum intensity projections.

### Laser ablation

Laser ablation was performed with the 40X objective using single pulses of the 3rd harmonic (355 nm) of a Q-switched Nd:YAG laser (5 ns pulse-width, Continuum Minilite II, Santa Clara, CA). Laser pulse energies ranged from 0.5 μJ to 10 μJ, depending on the experiment. A separate computer-controlled mirror and custom ImageJ plug-in were used to aim and operate the ablation laser so that ablation could be performed without any interruption to live imaging. The frame during ablation was retroactively considered t = 0 s.

### ImageJ radius analysis

The radius of plasma membrane damage was determined as previously described [14,15]. Briefly, the first frame of visible GCaMP6m fluorescence after wounding was used to determine the radius of calcium entry as a proxy for plasma membrane damage. The ImageJ Radial Profile Angle Plot plug-in was used on this image to determine the GCaMP intensity profile as a function of distance from the center of the wound. A custom MATLAB script was then used to determine the distance from the wound at which the intensity dropped to half its maximum. This distance corresponds to the radius of the radially-averaged calcium wave. This same method was applied to each frame in the first 30 seconds following a wound to find the radius of the “first calcium expansion” radius, determined as the maximum radius of GCaMP6m fluorescence within this time-frame.

The radii of all other zones of damage were found by drawing a circular region of interest in ImageJ, then using the “measure” tool in ImageJ to find the area. The radius was calculated based on A = π r^2^. To reduced bias, each dual-channel image was split into separate single-channel images before measuring, ensuring the size of the zone of damage in the red channel does not influence the measurement of the zone of damage in the green channel and vice versa. The zone of “laser-induced rupture” was drawn around the area that has clearly lost mCherry.NLS fluorescence compared to the surrounding punctate nuclei, by the second frame after wounding. The zone of “delayed cell lysis” was drawn around the area that had completely lost all mCherry.NLS fluorescence by 90 seconds following wounding. The zone of “nuclear membrane damage” was drawn around the area that had lost punctate mCherry.NLS fluorescence by 90 seconds following wounding, but still retained diffuse mCherry signal within the cell. The zone of “Ecadherin loss” was drawn around the ring of Ecadherin-GFP that remained around the center of the wound, connected to fully intact cells at 5 minutes after wounding. The zone of “chromatin disruption” was drawn around the zone where Histone-GFP punctae had become disorganized from the rest of the epithelial sheet. Although measurement of this zone was subjective, in many samples there was a clear delineation between Histone-GFP fluorescence that was disorganized and clumped in the center of the wound compared to organized nuclei that remained intact surrounding the wound.

### Equations and confidence intervals

The linear correlations, including confidence intervals and prediction bands, were found using Microsoft Excel and confirmed in Graphpad Prism. Briefly, the “LINEST” function in Microsoft Excel was used to calculate the linear regression values for the eleven equations presented in Figs 2–5, representing Eqs 1–11, as well as their inverse functions where the dependent and independent variables were swapped, representing Eqs 12–22 (S1 Dataset). Eqs 1–22 were plotted in Graphpad Prism, with the 95% confidence intervals and 95% single-prediction bands determined automatically in the software. The equations to yield the 95% confidence intervals (i.e., predicted value +/- CI_95%_) and 95% single-prediction bands (predicted value +/- SPB_95%_) were confirmed to be the following, as described previously [51,52]:

$${CI}_{95\%}=t_{crit}*s_{res}*\sqrt{\frac{1}{n}+\frac{{\left(x-\bar{x}\right)}^{2}}{\sum {\left(x_{i}-\bar{x}\right)}^{2}}}$$

and

$${SPB}_{95\%}=t_{crit}*s_{res}*\sqrt{1+\frac{1}{n}+\frac{{\left(x-\bar{x}\right)}^{2}}{\sum {\left(x_{i}-\bar{x}\right)}^{2}}}$$

Where t_crit_ is the critical t value for α = 0.05, s_res_ is the standard error of the predicted y for each x is the root-mean-square error of the measured versus predicted y-values, n is the sample size, x is the value of x where the confidence intervals are evaluated, x is the average value of x.

To estimate the extent of one region of damage from any other given region of damage (e.g. Fig 6), some equations were combined together. For example, to determine the radius of the first calcium expansion from a given radius of laser-induced rupture, Eq 3 was combined with Eq 7 to yield a new Eq 23:

$$PM=m_{1}*LR+b_{1}$$ (Eq 3)

$$1st=m_{2}*PM+b_{2}$$ (Eq 7)

yields:

$$1st=m_{2}*m_{1}*LR+m_{2}*b_{1}+b_{2}$$ (Eq 23)

This method was used to derive Eqs 23–42, using the most efficient “path” from one known value to one unknown value. In the cases where two combinations of equations were equally valid “paths”, then the two combinations were averaged together. For example, to determine the radius of the Ecadherin loss from a given radius of laser-induced rupture, Eqs 1 and 8 can be combined or Eqs 2 and 9 can be combined. In this case, both were averaged together.

To determine the 95% confidence intervals of Eqs 23–42, the uncertainties from each equation were propagated as described previously [53]. Briefly, the partial derivative with respect to each slope and intercept in the final equation was multiplied by the respective 95% confidence interval of the slope or intercept, and these were then summed in quadrature. Using y and x to represent a generic two-step path to calculate y given x, one can calculate the confidence interval of the predicted y-value at each x using:

$${CI}_{y,95\%}\approx \sqrt{{{\left(\frac{\partial y}{\partial m_{1}}\right)}_{x}}^{2}\sigma_{m_{1}}^{2}+{{\left(\frac{\partial y}{\partial m_{2}}\right)}_{x}}^{2}\sigma_{m_{2}}^{2}+{{\left(\frac{\partial y}{\partial b_{1}}\right)}_{x}}^{2}\sigma_{b_{1}}^{2}+{{\left(\frac{\partial y}{\partial b_{2}}\right)}_{x}}^{2}\sigma_{b_{2}}^{2}}$$

Where each partial derivative is evaluated at x, $\sigma_{m_{i}}^{2}$ is the 95% CI of the slope *m_i_* and $\sigma_{b_{i}}^{2}$ is the 95% CI of the intercept *b_i_*.

This process yielded the 42 linear equations designed to approximate any of the seven zones of damage from a measurement of any single zone. The supporting information includes an Excel dataset that contains all necessary regression information for all 42 equations (S2 Dataset). Further, it includes individual sheets where a single known radius value can be input for one zone of damage, and the approximate radius and 95% confidence intervals for the other six unknown zones of damage will be automatically calculated (S2 Dataset).

## Supporting information

S1 Fig Some cells with nuclear membrane damage recover. Timelapse depicting diffuse mCherry.NLS signal from the region of nuclear membrane damage at 60 minutes returning to nuclei and becoming punctate at 90 minutes post-wound (arrowheads). This process occurs progressively over the course of 2–3 hours after wounding and the arrowheads represent a small selection of the nuclei around a wound that undergo this behavior throughout the repair process. Scale bar = 50 μm.
(PDF)

S2 Fig The region of Ecadherin loss expands over 90 minutes. Timelapse of Ecadherin-GFP (green) and pnr>mCherry.NLS (magenta) following wounding, from S1 Movie. Immediately after wounding (0 min), Ecadherin is lost from the center of the wound and looks substantially similar by 5 min after wounding, as analyzed in Fig 4; by 90 minutes, the region of Ecadherin loss is much larger. During these ~90 minutes, Ecadherin-GFP appears to be gradually lost evenly across the region of delayed cell lysis (red circle), which was estimated by the loss of mCherry.NLS at 5 min. Cells that appear intact at 0 and 5 min but eventually lose Ecadherin have clearly died (labeled with yellow asterisks at 0 and 90 min). The region of nuclear membrane damage (blue circle) is evident by mCherry.NLS escaping from the nucleus immediately after wounding. Some cells with nuclear membrane damage recover whereas those closer to the center die, as illustrated by their recovering the mCherry.NLS signal and maintaining Ecadherin-GFP. n = 5 pupae. Scale bar = 50 μm.
(PDF)

S1 Movie The region of Ecadherin loss expands over 90 minutes. Timelapse movie of Ecadherin-GFP (green) and pnr>mCherry.NLS (magenta) over 90 minutes following wounding. Movie shows Ecadherin gradual loss in cells that initially appeared intact.
(MP4)

S1 Dataset Minimal data set containing the radius determined for each sample used in this study, organized by Figure. This dataset also includes the values for the linear regression analyses used to derive Eqs 1–22.
(XLSX)

S2 Dataset Data set containing Eqs 1–42, which can be used to approximate any zone of damage from a single known zone. Sheet “Total Equations List” lists all 42 equations with the necessary information to derive confidence intervals when using these equations. The other seven sheets allow the user to enter a single known radius value for one of the respective zones of damage in cell “B1”, and the approximate radii of the remaining six zones of damage will be automatically calculated in cells “B4–B9”, with 95% confidence intervals and expected ranges automatically calculated in cells “C4–E9”.
(XLSX)
