# Supplementary figures and images for: Zones of cellular damage around pulsed-laser wounds
Source: PLoS One. 2021 Sep 27;16(9):e0253032. doi: 10.1371/journal.pone.0253032 (PMC8476025; doi:10.1371/journal.pone.0253032)

mCherry-NLS

60 min

70 min

80 min

90 min

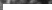

Supplement: S1 Fig — Timelapse depicting diffuse mCherry.NLS signal from the region of nuclear membrane damage at 60 minutes returning to nuclei and becoming punctate at 90 minutes post-wound (arrowheads). This process occurs progressively over the course of 2–3 hours after wounding and the arrowheads represent a small selection of the nuclei around a wound that undergo this behavior throughout the repair process. Scale bar = 50 μm. (PDF) [file pone.0253032.s001.pdf]

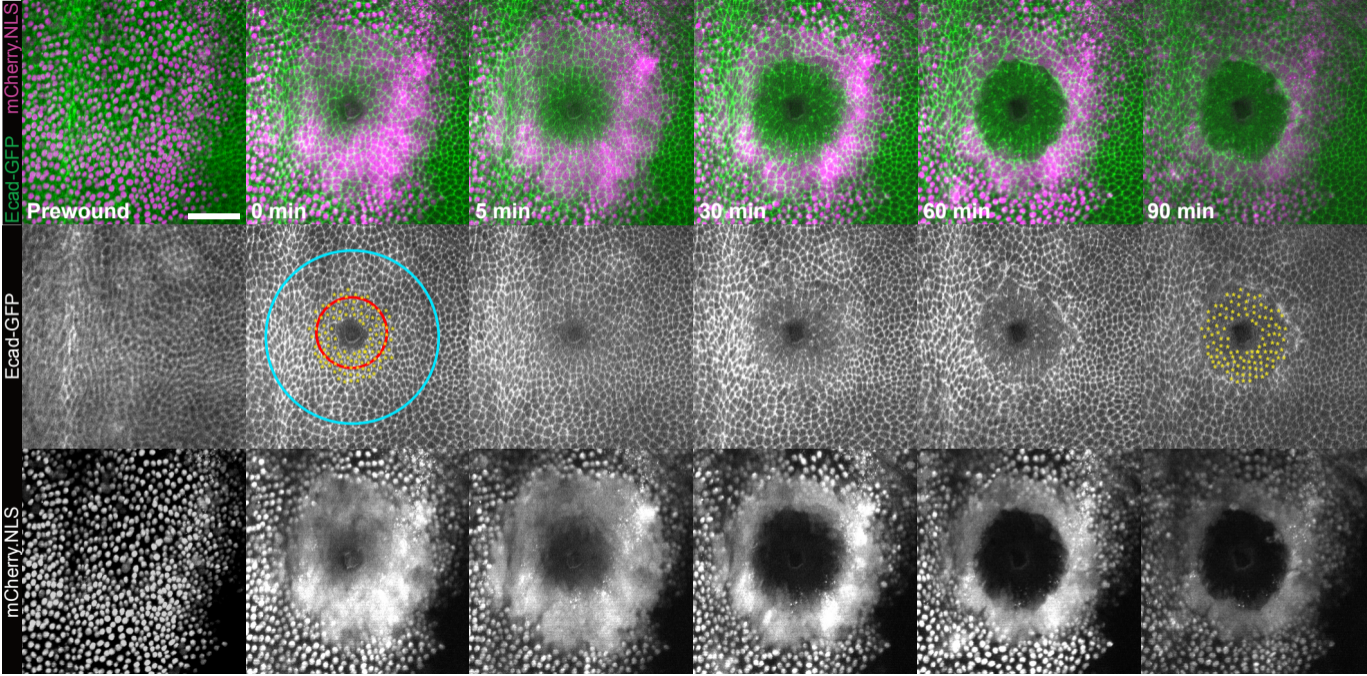

Supplement: S2 Fig — Timelapse of Ecadherin-GFP (green) and pnr>mCherry.NLS (magenta) following wounding, from S1 Movie. Immediately after wounding (0 min), Ecadherin is lost from the center of the wound and looks substantially similar by 5 min after wounding, as analyzed in Fig 4; by 90 minutes, the region of Ecadherin loss is much larger. During these ~90 minutes, Ecadherin-GFP appears to be gradually lost evenly across the region of delayed cell lysis (red circle), which was estimated by the loss of mCherry.NLS at 5 min. Cells that appear intact at 0 and 5 min but eventually lose Ecadherin have clearly died (labeled with yellow asterisks at 0 and 90 min). The region of nuclear membrane damage (blue circle) is evident by mCherry.NLS escaping from the nucleus immediately after wounding. Some cells with nuclear membrane damage recover whereas those closer to the center die, as illustrated by their recovering the mCherry.NLS signal and maintaining Ecadherin-GFP. n = 5 pupae. Scale bar = 50 μm. (PDF) [file pone.0253032.s002.pdf]
